# A multiple indicators multiple causes model of late-life depression in Latin American countries

**DOI:** 10.1016/j.jad.2015.05.053

**Published:** 2015-09-15

**Authors:** Anamaria Brailean, Mariella Guerra, Kia-Chong Chua, Martin Prince, Matthew A. Prina

**Affiliations:** a Department of Health Service and Population Research, Institute of Psychiatry, Psychology and Neuroscience, King's College London, London, UK; b Institute of Memory, Depression and Disease Risk, Avda Constructores 1230, Lima 12, Peru; c Peruvian University, Cayetano, Heredia, Lima, Peru

**Keywords:** MIMIC, multiple indicators multiple causes, MDD, major depressive disorder, DSM, diagnostic and statistical manual of mental disorders, PCA, principal components analysis, CFA, confirmatory factor analysis, GMS, geriatric mental state, CERAD, consortium to establish a registry for Alzheimer's disease, SEM, structural equation modelling, WLSMV, weighted least squares mean and variance-adjusted, CFI, comparative fit index, TLI, tucker lewis index, RMSEA, root mean square error of approximation, DIF, differential item functioning, Older adults, Depression, Cognitive function, Measurement invariance, Multiple indicators multiple causes model

## Abstract

**Background:**

The Euro-D depression scale consists of symptom clusters that may be differentially related to demographic and cognitive characteristics in older adults. This hypothesis needs further investigation and the role of measurement bias on substantive conclusions remains to be established.

**Method:**

The study sample comprised 10,405 community-dwelling older adults from six Latin American countries. We applied a Multiple Indicators Multiple Causes (MIMIC) model for a concurrent investigation of measurement bias and of the association between Euro-D symptom clusters and background variables.

**Results:**

The factorial validity of Euro-D, with a two-dimensional structure – affective suffering and motivation disturbance, was consistently supported in all countries. Although complete measurement invariance could not be assumed across countries, measurement bias was minor. Both Euro-D factors were unrelated to age, but related to gender, as well as to impairment in memory and verbal fluency. Gender differences were larger for affective suffering than for motivation disturbance, whereas differences in verbal fluency impairment were more strongly related to motivation disturbance.

**Limitations:**

Our analytic strategies could only examine invariance at the level of indicator thresholds. The generalisability of current findings needs to be examined in clinical populations. A wider set of cognitive tests is needed. We did not examine the compositional factors that could have accounted for the variation in Euro-D scores across countries, as this was beyond the aims of the paper.

**Conclusion:**

The current study adds evidence for the construct validity of Euro-D and for the possible differential association of depression symptom-clusters with gender and verbal fluency in older adults. An understanding of the heterogeneity of late-life depression may carry clinical implications for the diagnosis and treatment of depression in old age.

## Highlights

• The Euro-D factor structure was invariant across Latin American countries.
• Measurement bias was minor and did not alter substantive conclusions.
• Depression domains were differentially related to gender and cognitive function.
• Depression domains were unrelated to age.
• Cognitive function levels confounded the effect of age on depression domains.

## 1 Introduction

The clinical picture of late-life depression differs in several aspects from that observed in early-life depression. While Major Depressive Disorder (MDD) has a lower prevalence in older adults, subclinical depressive symptoms are more common in old age (Meeks et al., 2011; Romanoski et al., 1992). Moreover, older individuals are less likely to report depressed mood (Gallo et al., 1994) and more likely to report somatic symptoms, fatigue, appetite loss, concentration difficulties, lack of interest in activities and cognitive disturbance (Fountoulakis et al., 2003; Gallo and Rabins, 1999). Although core symptoms required for a standard diagnosis of Major Depressive Disorder include the presence of depressed mood or the loss of interest in activities, late-life depression may be characterized by a “depression without sadness” syndrome (Gallo and Rabins, 1999). As such, late-life depression may be under-detected and under-treated if clinical assessments focus uniquely on the MDD criteria of the Diagnostic and Statistical Manual of Mental Disorders, 4th Edition (DSM-IV).

The above findings suggest that factors other than depressed mood are likely to account for the high rates of subclinical depressive symptoms in old age. One such factor could be the presence of cognitive impairment, with older individuals showing pronounced executive function deficits, slower processing speed and concentration difficulties (Butters et al., 2004). It was suggested that ageing-related dysfunctions of fronto-striatal structures and cerebrovascular disease may precipitate late-life depressive symptoms (Alexopoulos et al., 2005; Alexopoulos et al., 2002). The clinical manifestations of these dysfunctions include executive function/verbal fluency impairment, as well as depression-like symptoms (e.g., reduced interest in activities).

Psychometric studies have provided factor analytic evidence that DSM-III symptoms of depression tend to cluster into two dimensions: disturbance of mood/affective suffering (e.g., depressed mood, tearfulness) and disturbance of motivation (e.g., lack of interest, poor concentration) (Forsell et al., 1994). Similar depression domains have been reported by factor analytic studies of Euro-D, a scale developed to assess symptoms of depression in older adults (Castro-Costa et al., 2007; Prince et al., 1999a; Verropoulou and Tsimbos, 2007). Furthermore, these studies showed that the two Euro-D domains were differentially associated with demographic variables and cognitive function levels. For instance, whereas females reported higher levels of affective suffering than males, this was not the case for motivation symptoms. This finding is consistent with a large body of literature that documented gender differences in mood disturbances (Djernes, 2006; Inaba et al., 2005; Mirowsky, 1996). Furthermore, while the severity of motivation symptoms increased with age, the severity of affective suffering symptoms did not. Also, a positive association was found between verbal fluency impairment and the severity of motivation symptoms, but not affective suffering symptoms. Memory impairment was unrelated to either symptom clusters. Taken together, these findings provided indirect support for the “depression-executive dysfunction hypothesis” which posits that late-life depression can present as motivation-related symptoms driven by ageing-related decline in executive function (Alexopoulos, 2005). Given that the distinction between affective suffering and motivation symptoms may carry clinical implications for the diagnosis and treatment of depression in old age, this hypothesis warrants further investigation.

The assessment of depressive symptoms across geographical regions requires instruments that are culturally-valid. Cultural beliefs can influence response behaviours leading to biased estimates of group differences in trait levels. An instrument is culturally invariant when individuals from different cultures have similar probabilities of item endorsement. A study that investigated the invariance of Euro-D across European countries suggested that the affective suffering factor is better characterised and more invariant across European countries than the motivation factor (Castro-Costa et al., 2008). Further investigation is needed to assess the validity of Euro-D in low and middle income countries.

This study aimed:

a. to establish the factor structure of Euro-D across six Latin American countries;
b. to determine whether measurement bias has weakened or exaggerated any differences in depression functioning between countries, gender, age and cognitive function levels;
c. to test previous hypotheses of a differential association of depression domains with age, gender, verbal fluency and memory performance.

## 2 Method

### 2.1 Participants

The study sample consisted of 10,405 older adults from six Latin American countries (Peru, Venezuela, Mexico, Puerto Rico, Cuba, Dominican Republic) who took part in the first wave of population-based surveys conducted by the 10/66 Dementia Research Group (Prince et al., 2007). All participants included in this study were at least 65 years old and had no diagnosis of dementia. Participants from Peru and Mexico were recruited from both urban and rural catchment areas, while participants from the other four countries were only recruited from urban areas. Studies were approved by local ethical committees in each country, and by the King's College London Research Ethics Committee. All individuals who took part in the surveys provided an informed consent. Interviews were conducted by trained individuals and were usually carried out in the interviewees' homes in a single session that lasted two to three hours.

### 2.2 Measures

The EURO–D (Prince et al., 1999b) is a scale developed from the Geriatric Mental State (GMS; Copeland et al., 1976) with the aim to assess 12 symptoms of late-life depression: depressed mood, pessimism, suicidality, guilt, sleep, interest, irritability, appetite, fatigue, concentration, enjoyment and tearfulness. Scores range from 0 to 12, with higher scores indicating greater symptom severity. Good internal consistency and criterion validity have been reported for this instrument (Prince et al., 1999b). A score of 4/5 or above has been reported as the optimal cut-off point for the identification of probable depression cases (Castro-Costa et al., 2007; Guerra et al., 2015). Principal component analysis (PCA) and confirmatory factor analysis have revealed that a two-factor solution-affective suffering and motivation-fits the data well across European countries (Castro-Costa et al., 2008; Prince et al., 1999a; Prince et al., 1999b), Latin American countries and India (Prince et al., 2004). Across European countries, stronger measurement invariance was found for the affective suffering factor than the motivation factor (Castro-Costa et al., 2008).

Assessments of cognitive function included the delayed recall of a 10-word list and the animal naming verbal fluency task adapted from the Consortium to Establish a Registry for Alzheimer's Disease (CERAD; Vanderhill et al., 2011). The delayed recall task required participants to recall 10 words that had been previously presented three times during the learning phase. The animal naming verbal fluency task required participants to name as many animals as possible over a period of 1 min. Performance on the verbal fluency task is thought to rely upon multiple cognitive processes such as semantic memory, language ability, and executive function components (Abwender et al., 2001; Henry and Phillips, 2006). Additionally, we used measures of age, gender and country of residence.

### 2.3 Statistical analysis

Structural Equation Modelling (SEM) analyses were conducted in MPlus Version 7.2 (Muthén and Muthén, 1998–2012) using mean and variance-adjusted weighted least squares (WLSMV) estimation. WLSMV is well suited for modelling categorical or ordered data and does not assume normally distributed variables (Brown, 2006). Confirmatory factor analysis was conducted to test a model with two first-order factors (affective suffering and motivation). Drawing upon previous factor analytic findings (Prince et al., 1999b), we hypothesised that loss of interest, lack of enjoyment and poor concentration should load on the motivation factor, whereas the other nine Euro-D items should load on the affective suffering factor. The model was tested in each Latin American country separately as well as in the pooled sample. Model fit was evaluated based on commonly adopted standards. Conventionally, a Chi-square index with a *P*-value above 0.05 shows good model fit, indicating a small discrepancy between the sample covariance matrices and the covariance matrices predicted by the model (Hu and Bentler, 1999). However, because Chi-Square statistic is sensitive to sample size, the model is nearly always rejected when large samples are used (Bentler and Bonett, 1980). Therefore, the comparative fit index (CFI; Bentler, 1990), and the Tucker Lewis index (TLI; Tucker and Lewis, 1973) were also used when evaluating model fit. Values above 0.90 were considered an acceptable fit, and above 0.95 a good fit. The root mean square error of approximation (RMSEA; Steiger, 1990) should have values below 0.10 for acceptable fit, and below 0.05 for good fit. Modification indices, which are derived from model Chi-square, were examined to decide whether additional parameters should be estimated to improve model fit.

After evaluating the measurement model, we proceeded to testing the validity of the model in the presence of covariates using Multiple Indicators Multiple Causes (MIMIC) modelling. MIMIC consists of a measurement model (established at the CFA stage), as well as a structural model. The structural model specifies the effect of the covariates/grouping variables on factors, thereby estimating group difference in latent factor means. The structural model can also include direct effects of the covariates on indicators, holding the latent variables constant. A significant direct effect indicates differential item functioning (DIF). DIF is present when response probabilities to an item differ between groups, despite the fact that groups have been matched for levels of the latent variables. For instance, if males have a lower probability than females of responding “Yes” to the item “Have you cried at all?”, despite similar levels of affective suffering, the item is considered to have gender DIF. The presence of DIF undermines measurement invariance. Conversely, measurement invariance is concluded when the probability of endorsing an item is comparable between groups, given similar levels of the latent trait score.

The following covariates were included in our model: country, gender, age, verbal fluency and delayed recall. Female gender was used as the reference group in all analyses. Dummy variables were created to allow for country comparisons and Cuba was used as the reference group as it has the largest sample size. A step-wise forward approach was used to assess the direct effects. To decide which direct path should be first added to the model, we examined the magnitude of the modification indices. Each modification index suggests how much the model could be improved by estimating an additional parameter (e.g., direct path). The modification index with the highest magnitude suggests the direct path that could be added to the model for the best improvement in model fit. Accordingly, we added the direct path with the highest modification index and compared this model with the simpler model which contained no direct path. A DIFFTEST (Muthén and Muthén, 1998–2012) was conducted to determine whether adding the direct path resulted in a significant improvement in model fit. Conventionally, a *χ*^2^ difference with a *P*-value below 0.05 indicates that the model that estimates the direct path fits the data better than the simpler model; therefore, the more complex model should be retained. Conversely, a *P*-value larger than 0.05 suggests that the estimation of the direct path does not result in a significant improvement in model fit; therefore, the simpler model should be retained. Direct paths can be added to the model until the inclusion of a new path does no longer result in a significant improvement in model fit. However, given our large sample size, DIFFTEST results are likely to be significant even when they reflect trivial improvement in model fit. Therefore, the number of direct paths included in our final model was determined after an examination of the practical impact of the DIFFTEST results. Specifically, we examined the magnitude of the direct effects, and the impact of the direct paths estimation on conclusions about group differences in factor means. Any improvement in model fit, albeit statistically significant, would have a trivial impact on our model results when the magnitude of the direct effects is very small and the size of the estimate of group differences in factor means remains largely unchanged. Furthermore, we examined the correlation between factor scores before and after adding each direct path. A correlation of almost one between factor scores suggests that the estimation of additional direct paths does not change the model in important ways.

## 3 Results

### 3.1 Descriptive statistics

Across variables, an overall 2.21% of data were missing. Descriptive statistics per country and in the overall sample are presented in Table 1. The overall sample consisted of a female majority, had a mean age of 74 years, an average score of 4.7 on delayed recall and an average score of 15.8 on verbal fluency. Country-specific proportions of individuals reporting the 12 Euro-D items are also presented in Table 1. Mean Euro-D scores varied from 1.7 in Puerto Rico to 2.9 in Dominican Republic. The proportion of EURO–D scores with a value of 4 or above varied from 16.3% in Puerto Rico to 36.4% in Dominican Republic (Castro-Costa et al., 2007).

### 3.2 Confirmatory factor analysis

Confirmatory factor analysis was applied to the pooled sample from all countries to test a model with 2 first-order factors. Table 2 shows the country-level and pooled sample CFA results, including goodness-of-fit indices, factor loadings and factor correlations. This measurement model showed good fit in each of the countries as well as in the pooled sample (CFI=0.964; TLI=0.955; RMSEA=0.005). In general, Euro-D items loaded well on the hypothesized factors. The factor correlation was *r*=0.66 in the overall sample and it ranged from *r*=0.55 in Peru to *r*=0.77 in Cuba.

### 3.3 Multiple Indicators Multiple Causes (MIMIC) model

**Fig. 1:**
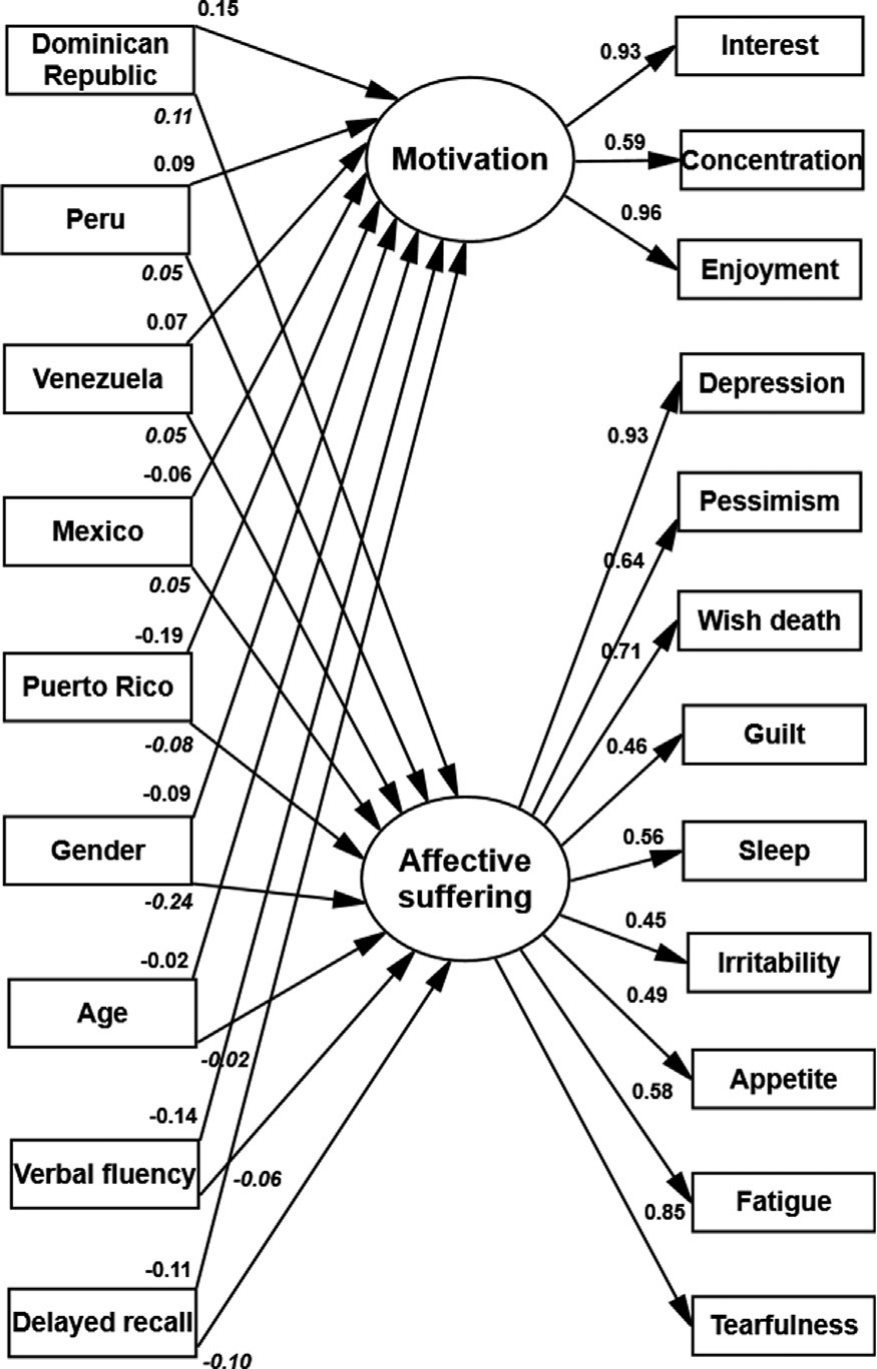
Multiple Indicator Multiple Causes (MIMIC) model showing the impact of background variables on the two factors, before adjusting for direct effects. Residual covariances are not shown in the model. For gender the reference group is female; for country the reference group is Cuba.

After adding the covariates, model fit declined but remained within acceptable ranges and factor loadings remained strong and significant (see Table 2 and Fig. 1). Modification indices suggested that model fit could be improved by freely estimating certain direct effects between the covariates and the indicators. We started by adding to the model the direct path with the highest potential to improve model fit and compared this model with the simpler model which contained no direct paths. A step-wise forward procedure was implemented until 10 direct paths between items and covariates were estimated (see Table 3). Nine of the 10 direct paths indicated differences in response behaviour across countries, the majority of which highlighted differences between Cuba and Peru. One direct path was related to gender, with males being more likely to report “irritability” than females. Most of the direct paths involved affective suffering items; only one direct path involved a motivation item (i.e., concentration). DIFFTEST results indicated a significant drop in *χ*^2^ for each additional direct path estimated. The magnitude of all direct effects was small (see Table 3 for standardized coefficients). We examined whether the estimation of each direct path led to changes in the size of the estimate of group differences in factor means. Specifically, we compared results of a model with no direct paths with a model with 5 direct paths and a model with 10 direct paths (see Table 4). The size of the estimates of group differences in factor means remained largely (e.g., males versus females) similar when estimating additional direct paths (see Table 4). Furthermore, an almost perfect correlation was found between factor scores derived before and after adding each direct path (e.g., adding the direct path from the country covariate “Peru” to the item “pessimism” resulted in a correlation of *r*=1.000, *P*<0.0001 for affective suffering, and *r*=0.991, *P*<0.0001 for motivation). Taken together, these findings suggest that any bias due to differential item functioning is minimal and that accounting for it has trivial consequences on model results. Accordingly, although the estimation of additional direct paths could have resulted in an additional improvement in model fit, we decided to limit our final model to 10 direct paths.

The effects of the covariates on latent mean scores are presented as unstandardised and standardised coefficients in Table 4. For the model with no direct paths, when examining country differences in affective suffering levels, we found that Dominican Republic, Peru, Venezuela and Mexico had significantly higher scores than Cuba, while Puerto Rico had significantly lower scores than Cuba. For motivation disturbance levels, we found that, compared to Cuba, scores were significantly higher in Dominican Republic, Peru and Venezuela, while they were significantly lower in Mexico and Puerto Rico. Gender differences were also found, with female participants having significantly higher levels of both affective suffering and motivation than male participants. The magnitude of the gender differences was larger for affective suffering (*β*=−0.24) compared to motivation (*β*=−0.09). Age did not have a significant effect on either motivation or affective suffering levels. Participants with higher levels of verbal fluency had significantly lower affective suffering (*β*=−0.06) and motivation disturbance levels (*β*=−0.14). Participants with higher levels of delayed recall had lower levels on both affective suffering (*β*=−0.11) and motivation (*β*=−0.10).

### 3.4 Sensitivity analysis

At the CFA stage modification indices suggested that model fit could be improved by estimating several more parameters. However, given that our initial measurement model had a good fit, we decided not to add additional parameters to the model. Our decision was motivated by the rationale that simpler models are more parsimonious and more likely to be replicated in different datasets (Crowley and Fan, 1997). However, to check the robustness of our conclusions, we performed a sensitivity analysis by estimating additional parameters (e.g., correlating residuals between “depression” and “tearfulness” items). This resulted in a slight improvement in model fit but did not alter model results regarding factor loadings, DIF effects, or the magnitude and direction of group differences in factor means. Similarly, at the MIMIC stage we decided to stop the step-wise forward estimation after the first 10 direct paths were added to the model. This decision was motivated by our findings suggesting a trivial impact on model results despite a statistically significant improvement in model fit.

Our results showed that age was not significantly related to either affective suffering or motivation. However, previous studies (Castro-Costa et al., 2007; Forsell et al., 1994) have suggested that the effect of age on motivation may be confounded by cognitive function levels. To test this hypothesis, we conducted post hoc analyses where we eliminated cognitive variables from our MIMIC model. When eliminating only the memory variable from the model, the effect of age on both affective suffering (*β*=0.03; *P*=0.80) and motivation (*β*<0.01; *P*=0.98) remained non-significant^1^. When eliminating only the verbal fluency variable, the effect of age on both affective suffering (*β*=−0.09; *P*=0.43) and motivation (*β*=−0.05; *P*=0.74) remained non-significant. When eliminating both cognitive variables from the analysis, older age was related to significantly higher levels of motivation disturbance (*β*=0.39; *P*<0.05) and affective suffering (*β*=0.26; *P*<0.05). Albeit statistically significant, the effect of age on Euro-D factors was small and it should be noted that we did not correct for multiple comparisons in our analyses. There is hence some support that cognitive function levels may confound the effects of age on both affective suffering and motivation.

## 4 Discussion

Using data from population-based surveys our study adds evidence for the construct validity of Euro-D in Latin American countries, a world region that faces unprecedented rates of demographic ageing and growing ageing-related health care costs (Alzheimer’s Disease International, 2009). Hypotheses were tested using an analytic strategy where the influence of any measurement bias would have been adjusted for and where the ordinal nature of item responses was appropriately accounted for.

We found support for previous findings that depression, as measured by Euro-D, can be interpreted in terms of two domains: affective suffering and motivation (Castro-Costa et al., 2007; Prince et al., 1999a; Prince et al., 1999b). In contrast to the study by (Castro-Costa et al., 2008) which suggested that the affective suffering factor had stronger measurement invariance than the motivation factor across European countries, our results indicate that Latin American countries differ more in their response behaviour to affective suffering items than motivation items. Moreover, we found that males are more likely to report irritability than females, in the absence of genuine gender differences in affective suffering levels. However, measurement non-invariance was not substantial. This conclusion was guided by findings of weak direct effects, as well as almost perfect correlations between factor scores derived before and after having added the direct effects. Also, adjusting for direct effects did not alter our conclusions about group differences in factor means. Taken together, these findings suggest that Euro-D has good construct validity and can be appropriately used for cross-cultural comparisons, as well as across age groups, gender and levels of cognitive impairment.

Our findings regarding gender differences are in line with previous studies that reported significantly higher levels of affective suffering in female compared to male participants (Castro-Costa et al., 2007; Forsell et al., 1994; Prince et al., 1999a). Although we also found that females had higher levels of motivation than males, the magnitude of the gender difference was much larger for affective suffering.

In contrast to previous studies that reported a significant positive association between age and motivation, but not affective suffering (Castro-Costa et al., 2007; Prince et al., 1999a), our findings show that age was not a significant predictor of either motivation or affective suffering. Of note, individuals with dementia were excluded from our analysis, which was not possible in the SHARE study analysis (Castro-Costa et al., 2007). Current findings should be interpreted in the context where any age differences in motivation and affective suffering levels have been adjusted for the effect of cognitive function (as well as for the effect of other covariates). When cognitive variables were excluded from our model, age became significantly related to both affective suffering and motivation levels. This is in line with previous studies (Castro-Costa et al., 2007; Forsell et al., 1994) which suggested that cognitive function levels may confound the effect of age on depression dimensions.

Better performance on verbal fluency and delayed recall tasks was significantly but weakly related to lower levels of both affective suffering and motivation symptoms. However, in line with findings by Castro-Costa et al. (2007), our study shows a stronger relative magnitude of the association between verbal fluency and motivation disturbance, compared to affective suffering.

## 5 Limitations

In the current study we opted for MIMIC modelling because this method allows for the concurrent investigation of the effect of multiple variables, measured categorically or continuously, on the factor model. Although MIMIC modelling is a robust method in the detection of non-invariance at the level of factor means and indicator intercepts, this method has its limitations. For instance, MIMIC modelling can only detect group differences in item thresholds (uniform DIF), but not group differences in item discrimination (non-uniform DIF) (Woods et al., 2009). Thus, our study assumed group differences in response behaviour that are constant across levels of affective suffering or across levels of motivation. Future investigation could explore whether inconsistencies in response behaviour occur at high/low levels of affective suffering/motivation by using an alternative method: multi-group factor analysis. A second limitation is that our findings are only relevant to general community-dwelling populations without probable dementia. Further research is needed to examine the generalisability of current findings to clinical populations. Another limitation is that our conclusions were based only on two measures of cognitive function (i.e. verbal fluency and delayed recall). A wider set of cognitive tests would be needed for a more comprehensive understanding of the differential associations between cognitive function and depressive symptoms in the elderly. Also, the question of whether there is a “depression without sadness” syndrome in the elderly has to be addressed beyond the cross-sectional context of the current investigation. Last but not least, the question of what compositional factors might be accounting for the variation in Euro-D scores and in the prevalence of depression cases across countries remains unexplored in the present paper. This question is worth addressing in future studies with a broader framework of variables that could explain the differences in depressive symptoms across countries. The primary aim of our paper was to investigate the validity of Euro-D and to examine group differences in depression domains. Our study has identified, to some extent, what is not contributing to the difference in Euro-D scores and in the prevalence of depression cases across countries (i.e. measurement bias/differential item functioning).

## 6 Conclusions

The current study extends previous investigations in several ways. First, our findings add support for the cross-cultural validity of Euro-D depression scale in Latin American countries. Second, the present study provides support for previous findings that Euro-D domains may be differentially associated with cognitive function levels and demographic characteristics in older adults. Greater severity of both affective suffering and motivation was related to female gender and to higher impairment in verbal fluency and memory. Gender differences were larger for the affective suffering factor, whereas individual differences in verbal fluency were more strongly associated with the motivation factor. Age was unrelated to either depression domain when cognitive function levels were controlled for. When cognitive function levels were not adjusted for, older age was related to both affective suffering and motivation disturbance. Third, we found that measurement bias was minor and did not alter substantive conclusions about the association of depression domains with demographic characteristics and cognitive function levels.

## Role of funding source

The funding sources had no involvement in the conduct of research or in the preparation of the article.

## Conflict of interest

none.

## Figures and Tables

**Table 1:** Demographic characteristics, cognitive function, and Euro-D item responses by country and overall sample.

	Cuba (*n*=2358)	DR (*n*=1592)	Peru (*n*=1589)	Venezuela (*n*=1638)	Mexico (*n*=1640)	Puerto Rico (*n*=1588)	Total (*n*=10,405)
Female (%)	64.6	65.2	60.1	63.9	62.7	67.5	64.0
Age	74.3(6.7)	74.5(7.1)	74.1(7.0)	72.0(6.5)	73.6(6.2)	75.4(6.8)	74.0(6.8)
Verbal fluency	16.6(5.9)	13.8(4.7)	17.0(5.4)	18.3(6.4)	14.8(4.9)	14.2(4.3)	15.8(5.6)
Delayed recall	5.1(1.9)	4.2(1.9)	4.7(2.0)	5.1(2.1)	4.3(1.9)	4.4(2.0)	4.7(2.0)
							
Euro-D symptoms (%)							
Depression	40	50	44	39	40	39	42
Pessimism	25	22	14	24	28	11	21
Wish death	14	14	8	8	13	8	11
Guilt	3	5	10	5	8	7	6
Sleep	34	39	22	35	27	23	30
Interest	8	17	10	9	6	3	9
Irritability	18	20	34	26	24	14	22
Appetite	9	19	11	10	13	9	12
Fatigue	17	35	33	30	28	20	26
Concentration	8	15	23	19	12	6	13
Enjoyment	8	18	7	6	5	2	8
Tearfulness	22	39	32	30	33	25	30
							
*Total Euro-D*	2.0(2.3)	2.9(2.6)	2.5(2.2)	2.4(2.3)	2.3(2.2)	1.7(2.0)	2.3(2.3)
*Depression case Cut-off score*≥*4 (%)*	22.6	36.4	27.3	26.3	26.0	16.3	25.6

Note: Means and standard deviations are presented unless otherwise stated.

**Table 2:** Factor loadings, factor correlation and fit indices by country (CFA) and overall sample (CFA and MIMIC).

Euro-D items	Cuba (*n*=2357)	DR (*n*=1592)	Mexico (*n*=1640)	Venezuela (*n*=1638)	Puerto Rico (*n*=1588)	Peru *n*=1588	Overall sample (*N*=10,403)	Overall sample (MIMIC) (*N*=10,372)
*Affective suffering factor*								
Depression	0.98	0.88	0.94	0.97	0.97	0.92	0.93	0.93
Pessimism	0.73	0.54	0.60	0.51	0.76	0.61	0.62	0.64
Wishing death	0.78	0.76	0.62	0.61	0.71	0.68	0.71	0.71
Guilt	0.45	0.43	0.36	0.37	0.52	0.52	0.42	0.46
Sleep	0.55	0.62	0.52	0.57	0.53	0.55	0.56	0.56
Irritability	0.37	0.57	0.36	0.45	0.57	0.26	0.41	0.45
Appetite	0.58	0.51	0.40	0.42	0.48	0.50	0.50	0.49
Fatigue	0.66	0.61	0.53	0.52	0.52	0.47	0.57	0.58
Tearfulness	0.88	0.80	0.82	0.84	0.90	0.80	0.84	0.85
								
*Motivation factor*								
Interest	0.95	0.91	0.73	0.92	0.90	0.95	0.93	0.93
Concentration	0.59	0.47	0.55	0.59	0.65	0.60	0.56	0.59
Enjoyment	0.94	0.93	0.80	0.99	0.95	0.93	0.95	0.96
								
*Factor correlation*	0.77	0.66	0.73	0.68	0.72	0.55	0.68	0.66
								
*Model fit*								
*χ*^2^ (df)	378(53)	278(53)	251(52)	361(53)	250(53)	279(53)	1551(53)	3351(143)
RMSEA	0.051	0.052	0.048	0.060	0.048	0.052	0.052	0.047
(90% CI)	(0.046–0.056)	(0.046–0.058)	(0.042–0.054)	(0.054–0.065)	(0.042–0.054)	(0.046–0.058)	(0.050–0.054)	(0.045–0.048)
CFI	0.976	0.963	0.966	0.960	0.971	0.954	0.964	0.913
TLI	0.970	0.954	0.957	0.950	0.964	0.943	0.955	0.894

**Table 3:** Alternative MIMIC models with direct effects between covariates and items estimated in a stepwise process (*N*=10,372).

Model		*χ*^2^ (df)	Δ*χ*^2^ (df)	CFI	TLI	RMSEA (90% CI)	*B*	S.E.	*β*
1	No direct effects	3351 (143)		0.913	0.894	0.047 (0.045–0.048)			
2	+ Peru→Concentration	3229 (142)	155	0.916	0.897	0.046 (0.044–0.047)	0.672	0.055	0.231
3	+ Peru→Pessimism	3102 (141)	153	0.920	0.901	0.045 (0.044–0.046)	−0.567	0.046	−0.197
4	+ Peru→Sleep	2972 (140)	120	0.923	0.905	0.044 (0.043–0.046)	−0.532	0.043	−0.187
5	+ Peru→Wishing death	2873 (139)	105	0.926	0.907	0.044 (0.042–0.045)	−0.594	0.055	−0.207
6	+ Male→Irritability	2769 (138)	104	0.929	0.910	0.043 (0.041–0.044)	0.297	0.029	0.141
7	+ Concentration→Venezuela	2679 (137)	110	0.931	0.912	0.042 (0.004–0.044)	0.577	0.057	0.198
8	+ Fatigue→Venezuela	2590 (136)	122	0.933	0.915	0.042 (0.040–0.043)	0.454	0.043	0.161
9	+ Pessimism→Puerto Rico	2495 (135)	158	0.936	0.918	0.041 (0.040–0.042)	−0.510	0.046	−0.174
10	+ Depression→Peru	2403 (134)	86	0.938	0.920	0.040 (0.039–0.042)	−0.496	0.040	−0.171
11	+ Fatigue→Dominican Republic	2334 (133)	63	0.940	0.922	0.040 (0.039–0.041)	0.398	0.043	0.138

Notes: Δ*χ*^2^=DIFFTEST; *B*=unstandardised estimate; S.E.=standard error; *β*=standardised estimate; All *χ*^2,^, Δ*χ*^2^ and *β* values are significant at *P*<0.0001.

**Table 4:** Impact of country, gender, age, verbal fluency and delayed recall on the affective suffering and motivation factors.

	Model with no direct paths				Model with 5 direct paths				Model with 10 direct paths			
***Affective suffering***	*B*	S.E.	*P*-value	*β*	*B*	S.E.	*P*-value	*β*	*B*	S.E.	*P*-value	*β*
Dominican Republic	0.283	0.037	<0.001	0.105	0.281	0.037	<0.001	0.103	0.225	0.037	<0.001	0.081
Peru	0.143	0.036	<0.001	0.053	0.401	0.037	<0.001	0.148	0.607	0.043	<0.001	0.220
Venezuela	0.144	0.036	<0.001	0.054	0.144	0.036	<0.001	0.054	0.077	0.037	<0.05	0.028
Mexico	0.132	0.036	<0.001	0.049	0.132	0.036	<0.001	0.049	0.132	0.036	<0.001	0.048
Puerto Rico	−0.217	0.039	<0.001	−0.080	−0.216	0.039	<0.001	−0.079	−0.147	0.039	<0.001	−0.053
Gender	−0.477	0.024	<0.001	−0.235	−0.509	0.024	<0.001	−0.250	−0.514	0.024	<0.001	−0.248
Age	−0.030	0.002	0.124	−0.180	−0.030	0.002	0.119	−0.180	−0.030	0.002	0.115	−0.190
Verbal fluency	−0.011	0.002	<0.001	−0.061	−0.011	0.002	< 0.001	−0.061	−0.011	0.002	<0.001	−0.060
Delayed recall	−0.049	0.006	<0.001	−0.102	−0.049	0.006	< 0.001	−0.101	−0.049	0.006	<0.001	−0.100
Total variance explained	9%				11%				12%			
			
***Motivation***	*B*	S.E.	*P*-value	*β*	*B*	S.E.	*P*-value	*β*	*B*	S.E.	*P*-value	*β*
Dominican Republic	0.397	0.047	<0.001	0.146	0.396	0.047	<0.001	0.146	0.396	0.047	<0.001	0.146
Peru	0.245	0.051	<0.001	0.090	0.031	0.054	0.570	0.011	0.031	0.054	0.570	0.011
Venezuela	0.196	0.051	<0.001	0.073	0.195	0.051	<0.001	0.073	0.018	0.055	0.746	0.007
Mexico	−0.154	0.054	<0.01	−0.057	−0.155	0.054	<0.01	−0.058	−0.157	0.054	<0.01	−0.059
Puerto Rico	−0.519	0.062	<0.001	−0.190	−0.520	0.063	<0.001	−0.191	−0.522	0.063	<0.001	−0.192
Gender	−0.174	0.033	<0.001	−0.085	−0.174	0.033	<0.001	−0.086	−0.174	0.033	<0.001	−0.086
Age	−0.040	0.002	0.131	−0.240	−0.040	0.002	0.131	−0.240	−0.040	0.002	0.131	−0.250
Verbal fluency	−0.024	0.003	<0.001	−0.137	−0.024	0.003	<0.001	−0.137	−0.024	0.003	<0.001	−0.138
Delayed recall	−0.055	0.008	<0.001	−0.111	−0.055	0.008	<0.001	−0.112	−0.055	0.008	<0.001	−0.112
Total variance explained	12%				11%				12%			

Notes: For gender the reference group is female; for country the reference group is Cuba; *B* and *β* coefficients for age are presented per 10 years; verbal fluency coefficients are presented per animal named; delayed recall coefficients are presented per word recalled.

## Footnotes

1 *β* coefficients for age are presented per 10 years.

## References

[bib1] Abwender D.A., Swan J.G., Bowerman J.T., Connolly S.W. (2001). Qualitative analysis of verbal fluency output: review and comparison of several scoring methods. Assessment.

[bib2] Alexopoulos G.S. (2005). Depression in the elderly. Lancet.

[bib3] Alexopoulos G.S., Kiosses D.N., Heo M., Murphy C.F., Shanmugham B., Gunning-Dixon F. (2005). Executive dysfunction and the course of geriatric depression. Biol. Psychiatry.

[bib4] Alexopoulos G.S., Kiosses D.N., Klimstra S., Kalayam B., Bruce M.L. (2002). Clinical presentation of the “depression-executive dysfunction syndrome” of late life. Am. J. Geriatr. Psychiatry.

[bib5] Alzheimer’s Disease International, 2009. World Alzheimer Report, London.

[bib6] Bentler P.M. (1990). Comparative fit indexes in structural models. Psychol. Bull..

[bib7] Bentler P.M., Bonett D.G. (1980). Significance tests and goodness of fit in the analysis of covariance-structures. Psychol. Bull..

[bib8] Brown T. (2006). Confirmatory Factor Analysis for Applied Research.

[bib9] Butters M.A., Whyte E.M., Nebes R.D., Begley A.E., Dew M.A., Mulsant B.H., Zmuda M.D., Bhalla R., Meltzer C.C., Pollock B.G., Reynolds C.F., Becker J.T. (2004). The nature and determinants of neuropsychological functioning in late-life depression. Arch. Gen. Psychiatry.

[bib10] Castro-Costa E., Dewey M., Stewart R., Banerjee S., Huppert F., Mendonca-Lima C., Bula C., Reisches F., Wancata J., Ritchie K., Tsolaki M., Mateos R., Prince M.J. (2007). Prevalence of depressive symptoms and syndromes in later life in ten European countries: the SHARE study. Br. J. Psychiatry: J. Ment. Sci..

[bib11] Castro-Costa E., Dewey M., Stewart R., Banerjee S., Huppert F., Mendonca-Lima C., Bula C., Reisches F., Wancata J., Ritchie K., Tsolaki M., Mateos R., Prince M.J. (2008). Ascertaining late-life depressive symptoms in Europe: an evaluation of the survey version of the EURO-D scale in 10 nations. The SHARE project. Int. J. Methods Psychiatr. Res..

[bib12] Copeland J.R., Kelleher M.J., Kellett J.M., Gourlay A.J., Gurland B.J., Fleiss J.L., Sharpe L. (1976). A semi-structured clinical interview for the assessment of diagnosis and mental state in the elderly: the Geriatric Mental State Schedule. I. Development and reliability. Psychol. Med..

[bib13] Crowley S.L., Fan X.T. (1997). Structural equation modeling: basic concepts and applications in personality assessment research. J. Pers. Assess..

[bib14] Djernes J.K. (2006). Prevalence and predictors of depression in populations of elderly: a review. Acta Psychiatr. Scand..

[bib15] Forsell Y., Jorm A.F., Winblad B. (1994). Association of age, sex, cognitive dysfunction, and disability with major depressive symptoms in an elderly sample. Am. J. Psychiatry.

[bib16] Fountoulakis K.N., O’Hara R., Iacovides A., Camilleri C.P., Kaprinis S., Kaprinis G., Yesavage J. (2003). Unipolar late-onset depression: a comprehensive review. Ann. Gen. Hosp. Psychiatry.

[bib17] Gallo J.J., Anthony J.C., Muthen B.O. (1994). Age-differences in the symptoms of depression-a latent trait analysis. J. Gerontol..

[bib18] Gallo J.J., Rabins P.V. (1999). Depression without sadness: alternative presentations of depression in late life. Am. Fam. Physician.

[bib19] Guerra M., Ferri C., Llibre J., Prina A., Prince M. (2015). Psychometric properties of EURO-D, a geriatric depression scale: a cross-cultural validation study. BMC Psychiatry.

[bib20] Henry J.D., Phillips L.H. (2006). Covariates of production and perseveration on tests of phonemic, semantic and alternating fluency in normal aging. Neuropsychology, development, and cognition. Section B. Aging Neuropsychol. Cogn..

[bib21] Hu L.T., Bentler P.M. (1999). Cutoff criteria for fit indexes in covariance structure analysis: conventional criteria versus new alternatives. Struct. Equ. Model..

[bib22] Inaba A., Thoits P.A., Ueno K., Gove W.R., Evenson R.J., Sloan M. (2005). Depression in the United States and Japan: gender, marital status, and SES patterns. Soc. Sci. Med..

[bib23] Meeks T.W., Vahia I.V., Lavretsky H., Kulkarni G., Jeste D.V. (2011). A tune in “a minor” can “b major”: a review of epidemiology, illness course, and public health implications of subthreshold depression in older adults. J. Affect. Disord..

[bib24] Mirowsky J. (1996). Age and the gender gap in depression. J. Health Soc. Behav..

[bib25] Muthén, L.K., Muthén, B.O., 1998–2012. Mplus User's Guide, seventh edition, Los Angeles, CA.

[bib26] Prince M.J., Acosta D., Chil H., Copeland J., Dewey M., Scazufca M., Varghese M., Grp D.R. (2004). Effects of education and culture on the validity of the Geriatric Mental State and its AGECAT algorithm. Br. J. Psychiatry.

[bib27] Prince M.J., Beekman A.T., Deeg D.J., Fuhrer R., Kivela S.L., Lawlor B.A., Lobo A., Magnusson H., Meller I., van Oyen H., Reischies F., Roelands M., Skoog I., Turrina C., Copeland J.R. (1999). Depression symptoms in late life assessed using the EURO-D scale. Effect of age, gender and marital status in 14 European centres. Br. J. Psychiatry.

[bib28] Prince M.J., Ferri C.P., Acosta D., Albanese E., Arizaga R., Dewey M., Gavrilova S.I., Guerra M., Huang Y., Jacob K.S., Krishnamoorthy E.S., McKeigue P., Rodriguez J.L., Salas A., Sosa A.L., Sousa R.M.M., Stewart R., Uwakwe R. (2007). The protocols for the 10/66 dementia research group population-based research programme. BMC Public Health.

[bib29] Prince M.J., Reischies F., Beekman A.T., Fuhrer R., Jonker C., Kivela S.L., Lawlor B.A., Lobo A., Magnusson H., Fichter M., van Oyen H., Roelands M., Skoog I., Turrina C., Copeland J.R. (1999). Development of the EURO-D scale—a European, Union initiative to compare symptoms of depression in 14 European centres. Br. J. Psychiatry.

[bib30] Romanoski A.J., Folstein M.F., Nestadt G., Chahal R., Merchant A., Brown C.H., Gruenberg E.M., McHugh P.R. (1992). The epidemiology of psychiatrist-ascertained depression and DSM-III depressive disorders. Results from the Eastern Baltimore mental health survey clinical reappraisal. Psychol. Med..

[bib31] Steiger J.H. (1990). Structural model evaluation and modification-an interval estimation approach. Multivar. Behav. Res..

[bib32] Tucker L.R., Lewis C. (1973). A reliability coefficient for maximum likelihood factor analysis. Psychometrika.

[bib33] Vanderhill, S., Strauss, E., Sherman, E.M.S., 2011. Consortium to Establish a Registry on Alzheimer's Disease pp. 690–692

[bib34] Verropoulou G., Tsimbos C. (2007). Socio-demographic and health-related factors affecting depression of the Greek population in later life: an analysis using SHARE data. Eur. J. Ageing.

[bib35] Woods C.M., Oltmanns T.F., Turkheimer E. (2009). Illustration of MIMIC-model DIF testing with the Schedule for nonadaptive and adaptive personality. J. Psychopathol. Behav. Assess..

